# Tissue surface area and tumor cell count affect the success rate of the Oncomine Dx Target Test in the analysis of biopsy tissue samples

**DOI:** 10.1111/1759-7714.13743

**Published:** 2020-11-13

**Authors:** Daiji Nemoto, Tomoyuki Yokose, Kayoko Katayama, Shuji Murakami, Terufumi Kato, Haruhiro Saito, Masaki Suzuki, Daisuke Eriguchi, Joji Samejima, Takuya Nagashima, Hiroyuki Ito, Kouzo Yamada, Haruhiko Nakayama, Munetaka Masuda

**Affiliations:** ^1^ Department of Thoracic Surgery Kanagawa Cancer Center Yokohama Japan; ^2^ Department of Pathology Kanagawa Cancer Center Yokohama Japan; ^3^ Unit of Cancer Survivorship and Education Kanagawa Cancer Center Research Institute Yokohama Japan; ^4^ Department of Thoracic Oncology Kanagawa Cancer Center Yokohama Japan; ^5^ Department of Surgery Yokohama City University Yokohama Japan

**Keywords:** Biopsy, molecular diagnostic testing, next generation sequencing, non‐small cell lung cancer

## Abstract

**Background:**

The Oncomine Dx Target Test (ODxTT) is a next‐generation sequencing‐based companion diagnostic test which has been recently developed; however, its analysis success rate could be improved, especially for small samples. The aim of this study was to identify the pathological factors associated with biopsy specimens that affect the analysis success rate of ODxTT.

**Methods:**

We retrospectively investigated 119 cases subjected to ODxTT at Kanagawa Cancer Center. Data pertaining to the results of *BRAF* V600E mutation analysis in ODxTT and pathological factors based on microscope slides were collected. Pathological factors including tissue surface area, tumor cell count, and tumor content rate were assessed. We constructed receiver operating characteristic curves and determined the optimal cutoff values of each pathological factor. Multivariate logistic analysis was used to identify significant factors.

**Results:**

A total of 98 of 119 samples were successfully analyzed (75.6%). The tissue surface area and tumor cell count were significantly higher in the group associated with analysis success (*P* < 0.001 and *P* = 0.011, respectively), and their optimal cutoff values were 1.04 mm^2^ and 375 cells, respectively. A tissue surface area > 1.04 mm^2^ and tumor cell count >375 cells had a positive effect on the analysis success rate of ODxTT (odds ratio [OR] 0.10; 95% confidence interval [CI]: 0.03–0.35; *P* < 0.001 and OR 0.25; 95% CI: 0.07–0.90; *P* = 0.033, respectively).

**Conclusions:**

Selecting samples with a tissue surface area > 1.04 mm^2^ and a tumor cell count >375 cells might improve the analysis success rate of ODxTT.

**Key points:**

Significant findings of the study: We found that a tissue surface area > 1.04 mm^2^ and tumor cell count >375 cells had a positive effect on the analysis success rate of ODxTT in the analysis of biopsy tissue samples.

What this study adds: It is sometimes necessary to assess genetic alterations with a small biopsy sample in daily practice. The criteria mentioned above will help to determine which tests should be performed, ODxTT or multiple single‐gene testing.

## Graphical abstract

This study demonstrates that a tissue surface area >1.04 mm^2^ and tumor cell count >375 cells had a positive effect on the analysis success rate of the Oncomine Dx Target Test in the analysis of biopsy tissue samples. The criteria mentioned above will help to determine which tests should be performed; next‐generation sequencing‐based companion diagnostics or multiple single‐gene testing.

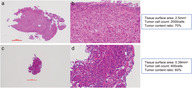

## Introduction

A large proportion of patients with non‐small cell lung cancer (NSCLC) are still being diagnosed at a locally advanced or metastatic stage.^1^, ^2^ Patients with advanced NSCLC are treated mainly using conventional chemotherapy and radiotherapy; however, since the 2000s, molecular target therapies have been developed as an effective option for these patients.^3^ Since the advent of the use of tyrosine kinase inhibitors for the treatment of patients with advanced NSCLC with epithelial growth factor receptor (*EGFR*) mutations, a new era has emerged in which various molecular therapeutic targets are being studied at the protein and gene levels. Because molecular targeted therapy provides a better overall survival or progression‐free survival to patients with specific gene alterations, such as *EGFR*, anaplastic lymphoma kinase (*ALK*), c‐ros oncogene 1 (*ROS1*), B‐Raf proto‐oncogene (*BRAF*), etc., the clinical treatment guidelines recommend the assessment of genetic alterations to stratify patients with advanced NSCLC.^4^

To establish a pathological diagnosis of advanced NSCLC, procedures that are less invasive than resection, such as transbronchial biopsy (TBB), endobronchial ultrasound‐guided transbronchial needle aspiration (EBUS‐TBNA), and computed‐tomography‐guided core needle biopsy (CTNB), are preferred. However, the biopsy specimens that are sampled using these procedures are frequently small. In turn, small specimens are disadvantageous in this setting, because each conventional sequential single‐gene test for companion diagnosis consumes the tissue samples. Because new molecular therapeutic drugs are being developed rapidly, an increasing number of target molecules will have to be tested in the near future, which will result in a further increase in tissue sample consumption.

The Oncomine Dx Target Test (ODxTT; Thermo Fisher Scientific, MA, US) is a next‐generation sequencing (NGS)‐based companion diagnostic for NSCLC that was approved by the US Food and Drug Administration (FDA) in 2017. Using as little as 10 ng of DNA or RNA, it can simultaneously evaluate 46 driver gene alterations. In April 2018, the test was approved as a companion diagnostic to detect the V600E mutation of *BRAF*, and in December 2018 it was granted reimbursement coverage in Japan. In February 2019, it was granted expanded approval for use in the detection of two types of *EGFR* mutation (exon 19 deletions and the L858R point mutation), as well as for *ALK* fusion and *ROS1* fusion genes. In October 2019, it was granted expanded approval for use in the detection of mutations of *EGFR* on exons 18 to 21.^5^ However, NGS‐based companion diagnostics sometimes fail to analyze gene alterations because of the insufficient quantity or quality of specimens; failure caused by insufficient quantity has been previously reported in 6.4% to 33.8% of cases.^6^, ^7^, ^8^ Although an improvement of the analysis success rate is needed in this setting, few studies have reported on the specimens that are suitable for NGS‐based companion diagnostics, including ODxTT.

In this study, we analyzed the characteristics of sample specimens to identify factors that improve the analysis success rate of ODxTT.

## Methods

### Study design

We retrospectively investigated information pertaining to the results of ODxTT, the procedures of tissue sampling, and the pathological factors of the tissue specimens. This retrospective study was approved by the Institutional Review Board of the Kanagawa Cancer Center (clinical registration no. 2020EKI‐16). The requirement for patient consent was waived because of the retrospective nature of the study.

### Patients

Among the patients with NSCLC who were pathologically diagnosed at the Kanagawa Cancer Center from June 2018 to September 2019, 119 cases who underwent ODxTT were investigated here. The procedures used for tissue sampling were determined by physicians according to the condition of the patients. The procedures included TBB, EBUS‐TBNA, endobronchial ultrasonography with a guide‐sheath (EBUS‐GS), and others (Table 1).

**Table 1:** Clinicopathological data of the 119 samples included in the current study

	Results of ODxTT		
	Success (*n* = 98)	Failure (*n* = 21)	*P*‐value
Tissue surface area (mm^2^, median, range)	1.65 (0.01–14.71)	0.62 (0.05–3.10)	<0.001
Tumor cell count (cells, median, range)	481.5(19–2933)	268.5 (9–1477)	0.011
Tumor cell rate (%, median, range)	30 (5–80)	45 (5–95)	0.121
Biopsy procedure			
TBLB with EBUS‐GS	68	15	0.068
EBUS‐TBNA	13	2	
TBLB	12	0	
Other	5	4	

The Mann–Whitney *U* test was used to analyze the mean difference of tissue surface area, tumor cell count, and tumor cell rate, while Fisher's exact test was used to analyze the proportion of the procedures of biopsy.

EBUS‐GS, endobronchial ultrasonography with a guide‐sheath; EBUS‐TBNA, endobronchial ultrasound‐guided transbronchial needle aspiration; ODxTT, Oncomine Dx Target Test; TBLB, transbronchial lung biopsy.

### Fixation and embedding of samples

Samples were fixed in 10% neutral buffered formalin solution for 6–24 hours and embedded with paraffin. A block of the sample is termed formalin‐fixed paraffin‐embedded (FFPE) tissue.

### Pathological diagnosis and evaluation of pathological factors

**Figure 1:**
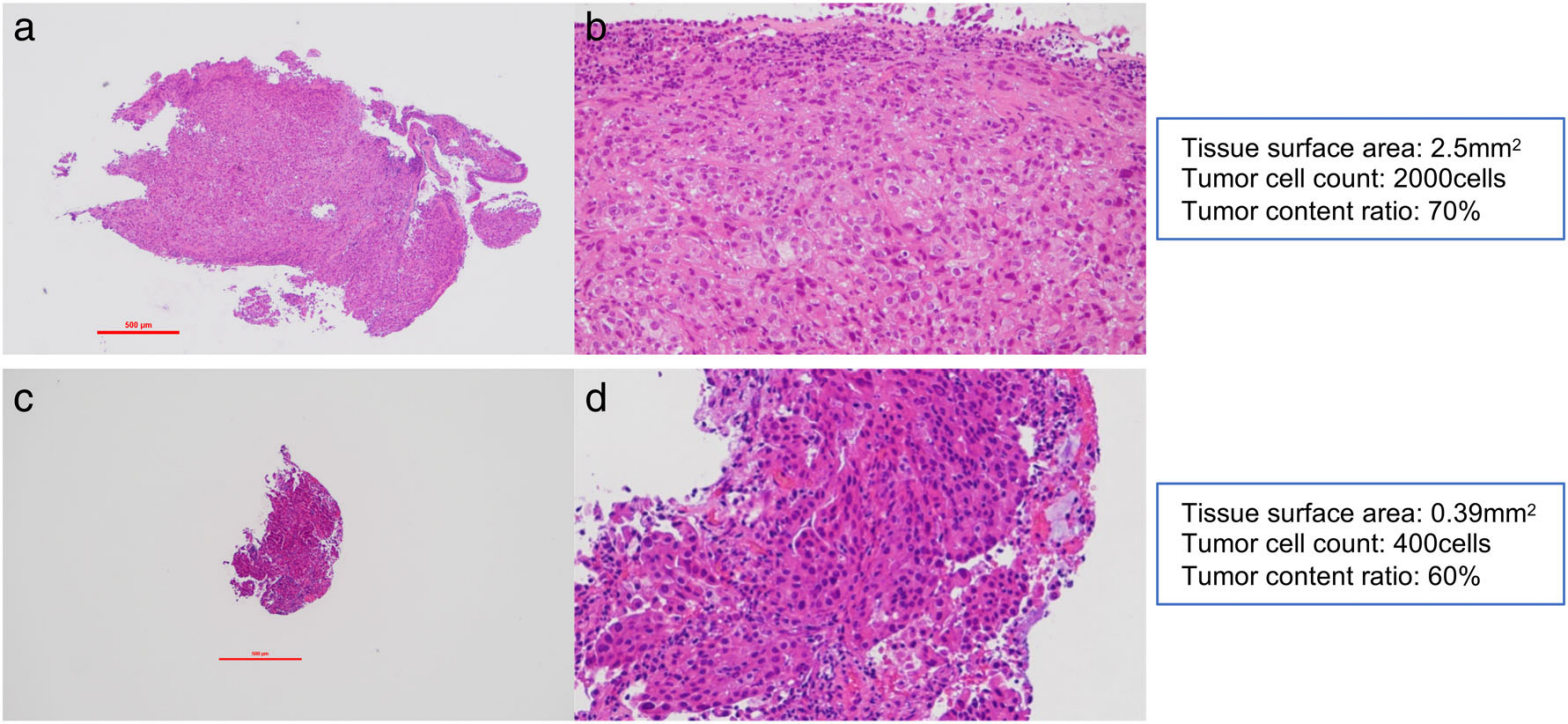
Pathological images of cases. (**a**, **b**) A case of analysis success. (**c**, **d**) A case of analysis failure. The red line scale in (a) and (b) indicates 500μm.

The pathological diagnosis was independently confirmed by pathologists with expertise in the diagnosis of thoracic diseases (MS and TY). Three pathological factors, ie, tissue surface area, tumor cell count, and tumor content rate, were evaluated. Tissue surface area (mm^2^) was defined as the area occupied by the tumor tissue on a slide and was measured using the NIS‐Elements D version 4.60 (Nikon, Japan) image analysis software. Tumor cell count (cells) was defined as the number of tumor cells on a slide and was estimated by multiplying the tissue surface area by the number of tumor cells per 1 mm^2^, as counted using an ocular micrometer. Tumor content rate (%) was defined as the percentage of tumor cells in the nucleated cells on a slide and was approximately rated in 5% increments by the pathologists (MS and TY). If the evaluations of the factors differed between the pathologists, a discussion was held between them and the factors were re‐evaluated. Pathological images of representative cases are shown in Figure 1.

### **ODxTT**

The ODxTT was performed for the clinical companion diagnosis of the V600E mutation of *BRAF*. The test was performed at central laboratory. Because ODxTT had been granted approval only as a companion diagnostic for *BRAF* V600E mutation in the study period in Japan, we investigated the results of the *BRAF* V600E mutation analysis by ODxTT exclusively.

The outline of the analytical method of ODxTT was as follows. First, DNA/RNA was extracted from 10 sections (5 μm thickness) of FFPE samples. The RNA was reverse transcribed to complementary DNA and a library was prepared. After preparing the template, sequencing was performed using an Ion PGM Dx Sequencer. Finally, the Torrent Suite Dx Software was used to assess the mapped reads at specific nucleotide locations and search for variation from the sequence information in the human reference sequence. The details of the analysis were based on the user guide.

In this study, we defined analysis “success” as cases that were successfully reported presence or absence of *BRAF* V600E mutation by ODxTT, and analysis “failure” as cases reported as “no call” and “invalid”.

### Statistical analysis

Variables were compared nonparametrically using the Mann–Whitney *U* test and Fisher's exact test for background analysis. To determine the optimal cutoff value of each pathological factor, we constructed receiver operating characteristic (ROC) curves. The area under the curve of ROC curves (AUC) was compared using the method described by DeLong *et al*.^9^ The optimal cutoff value of each factor was set at a value that maximized the Youden index. The pathological factors were divided into two groups based on the cutoff values obtained, and the correlation between the result of ODxTT and the pathological factor was analyzed using the chi‐squared test. We used a multivariate logistic regression analysis to identify factors that significantly affected the results of ODxTT, considering the pathological factors (divided into two groups) as explanation variables to be entered into the model.

Significance was set at *P* < 0.05. All statistical analyses were performed using EZR version 1.38 (Saitama Medical Center, Jichi Medical University, Saitama, Japan)^10^ which is a graphical user interface for R version 3.5.3 (The R Foundation for Statistical Computing, Vienna, Austria).

## Results

### Characteristics of samples

Factors pertaining to the specimens and sampling procedures are shown in Table 1. A total of 98 of the 119 samples were successfully analyzed (75.6%). The tissue surface area and tumor cell count were significantly higher in the analysis success group compared with the analysis failure group (1.65 *vs*. 0.62 mm^2^, *P* < 0.001 and 481.5 vs. 268.5 cells, *P* = 0.011, respectively).

### Optimal cutoff value of pathological factors

**Figure 2:**
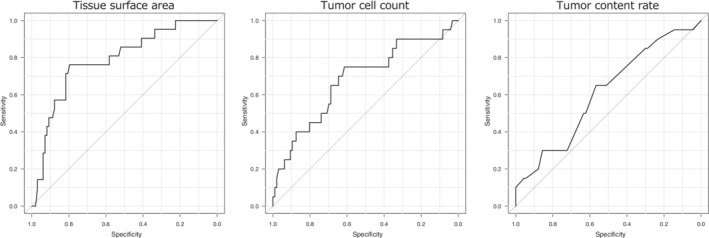
Receiver operating characteristic (ROC) curves of pathological factors.

The ROC curves of specimen factors are shown in Figure 2. The optimal cutoff values for tissue surface area, tumor cell count, and tumor content rate were 1.04 mm^2^, 375 cells, and 40%, respectively. The AUC of tissue surface area was significantly larger than that of tumor cell rate (*P* = 0.018). There were no significant differences between the other factor combinations. The subdivision of the pathological factors into two groups revealed that a tissue surface area above 1.04 mm^2^ and a tumor cell rate above 375 cells yielded a significantly high success rate of ODxTT (*P* < 0.001 and *P* = 0.006, respectively) (Table 2).

**Table 2:** Correlation between the results of the Oncomine Dx Target Test and pathological factors

		Results of ODxTT		
Factor		Success	Failure	*P*‐value
Tissue surface area (mm^2^)	>1.04	78	5	<0.001
	≦1.04	20	16	
Tumor cell count (cells)	>375	59	5	0.006
	≦375	37	15	
Tumor content rate (%)	>40	34	10	0.449
	≦40	56	10	

The factors were subdivided into two groups according to the optimal cutoff values obtained by the ROC curves: a tissue surface area above 1.04 mm^2^ and a tumor cell count above 375 had a significant effect on the test success rate.

ODxTT, Oncomine Dx Target Test.

**Table 3:** Multivariate logistic regression analysis of the factors that affected the results of the Oncomine Dx Target Test

	Results of ODxTT (0 = success/1 = failure)		
	OR	95% CI	*P*‐value
Tissue surface area (≦1.04 mm^2^/>1.04 mm^2^)	0.10	0.03–0.35	<0.001
Tumor cell count (≦375 cells/>375 cells)	0.25	0.07–0.90	0.033
Tumor cell rate (≦40%/>40%)	1.46	0.41–5.20	0.562

CI, confidence interval; ODxTT, Oncomine Dx Target Test; OR, odds ratio.

### Factors that significantly affected ODxTT results

The multivariate logistic regression analysis showed that a tissue surface area above 1.04 mm^2^ (odds ratio [OR], 0.10; 95% confidence interval [CI]: 0.03–0.35; *P* < 0.001) and a tumor cell count above 375 (OR, 0.25; 95% confidence interval [CI]: 0.07–0.90; *P* = 0.033) had a significantly positive effect on the success of ODxTT (Table 3).

## Discussion

Molecular targeted therapies provide a better prognosis for patients with advanced NSCLC compared with conventional chemotherapy. Improving the success rate of ODxTT will help the detection of gene alterations in NSCLC and guide the administration of adequate targeted therapies, even if the sampled specimen is small. To improve the success rate of ODxTT, the current study demonstrated that the tissue surface area and tumor cell count had an effect on the success rate of ODxTT using biopsy specimens from patients with NSCLC.

Before the advent of NGS‐based companion diagnostics, single‐gene testing was the only method of companion diagnosis of specific gene alterations. Single‐gene testing is also useful for detecting gene alterations; however, as the number of gene alterations to be tested simultaneously increase, a greater number of tissue slides is consumed. In addition, as the number of tested gene alterations increase, the success rate of reporting on the status of genetic alterations decreases.^11^ While targetable gene alterations for treatment are being actively studied and new molecular target drugs are being developed, less‐invasive biopsy procedures have not been developed sufficiently to increase dramatically the size of biopsy specimens. Thus, in some cases, testing all targetable gene alterations using sequential single‐gene testing is not feasible, and physicians are faced with the problem of which single‐gene tests should be prioritized. The use of NGS‐based companion diagnostics is one of the solutions. ODxTT is an NGS‐based test that investigates 46 gene alterations and is also available as companion diagnosis of four gene alterations (*EGFR*, *ALK*, *ROS1*, *BRAF*) in NSCLC in Japan.This comprehensive analysis consumes only a certain amount of sample tissue, in contrast to multiple single‐gene testing.

From the viewpoint of accuracy of ODxTT, the test result concordance rate between ODxTT and control methods had been examined at the time of FDA premarket approval. According to the results, the positive percent agreement/negative percent agreement/overall agreement of *BRAF* V600E, *EGFR*, and *ROS1* were 90.0/99.1/95.4%, 98.6/99.2/99.0%, and 85.0/100/97.8%, respectively. On the other hand, from the viewpoint of analysis success rate, 80 of 111 cases (72%) were not successfully analyzed due to no call or being invalid.^12^ In another study, the reported analysis failure rates of the investigational use of ODxTT were 30.8% for FNA and 24.6% for CTNB samples.^11^ The analysis failure rate in clinical practice at our institution (24.6%) is equivalent to the reported value. In brief, once the analysis is successfully performed, the result is almost reliable; however, the NGS‐based tests fail to analyze gene alterations at an adequate rate.

One of the main reasons for the failure of NGS‐based testing is insufficient sample quantity samples, which have reportedly varied from 6.4% to 33.8% in studies using NGS analyses other than ODxTT.^6^, ^7^, ^8^ No data are available to explain the failure of ODxTT analysis; moreover, to the best of our knowledge, no previous reports have addressed this issue. In Figure 1, there were relatively many ODxTT failures in “other” method (four of nine cases). All four of cases of analysis failure in the “other” procedure were biopsied by CTNB and their tissue surface area was lower than 1.04 mm^2^ (0.3–0.77 mm^2^). We considered that the factors of specimens rather than the procedure of biopsy were involved in the analysis failure.

Because NGS uses nucleic acids as materials, their quality and quantity affect the results of the analyses. It is important not only to maximize biopsy sample specimens, but also to optimize the preanalysis procedures, to ensure the quality and quantity of the samples.^13^ Kage *et al*. reported that the quality of DNA and RNA extracted from CTNB, EBUS‐TBNA, and TBB specimens is mostly adequate for targeted NGS analysis.^14^ Those authors used ddCq as a quality indicator of DNA and the percentage of RNA fragments above 200 nucleotides as a quality indicator of RNA; moreover, they demonstrated that, although the yield of nucleic acids was lower in biopsy versus resection specimens, the quality of the nucleic acids extracted from biopsy specimens was similar to that of resection specimens.^14^ Thus, proper sample processing may ensure the quality of the nucleic acids extracted from biopsy samples. Conversely, the size of biopsy specimens varies individually. The quantity of nucleotides cannot be detected before FFPE sample extraction; therefore, when dealing with a limited amount of tissue from patients with advanced NSCLC, a surrogate indicator that is detectable on microscope slides is needed. Morris *et al*. reported that the Paradigm Cancer Diagnostics test, which is a clinical‐grade NGS test run in a Clinical Laboratory Improvement Amendments‐certified and College of American Pathologists (CAP)‐accredited laboratory, could be performed using specimens as small as 10 mm^2^ with 5% tumor content without increasing low‐coverage genes and decreasing drug associations.^6^ The tissue surface area and tumor cell content were candidates for surrogate indicators.

In addition to the quality and quantity of NGS nucleic acids, other factors may affect the success or failure of NGS analysis. If an NGS analysis is expected to fail, it is not desirable to consume sample tissues for NGS‐based companion diagnostics. In such cases, rebiopsy is an option; alternatively, if the patient's medical condition does not permit rebiopsy, gene mutations can be searched using a sequential single‐gene test as much as possible. Therefore, whether a tissue sample is suitable for NGS‐based companion diagnostics must be determined on a microscope slide.

To our knowledge, the current study was the first to examine the factors on microscope slides related to the analysis success rate of ODxTT. Here, we demonstrated that the tissue surface area and tumor cell content (with optimal cutoff values of 1.04 mm^2^ and 375 cells, respectively) were positive predictive factors in this context. Using image analysis software, the measurement of the tissue surface area is relatively easy; therefore, we consider this parameter as being suitable for use in daily clinical practice. Conversely, although counting tumor cells is a little laborious, it is considered acceptable in daily clinical practice. We propose the use of samples that meet both or at least one of the criteria to improve the analysis success rate of ODxTT.

One of the limitations of the current study was that we analyzed only the results of *BRAF* V600E mutation analyses, that was detected by DNA sequencing, among the results of ODxTT. Because ODxTT is used worldwide for multiplex gene testing, it is also necessary to verify whether the tissue surface area affects the results of analyses of other gene alterations, especially gene alterations detected by RNA sequencing such as *ALK* fusion gene and *ROS1* fusion gene. Another limitation was that our study included cases with a lower tumor content ratio than 24%. According to the summary of safety and effectiveness data of FDA premarketing approval, limit of detection of BREF V600E was 12% of allele frequency,^12^ that corresponded to tumor content ratio of 24%. However, cases with lower tumor content ratio than 24% were successfully analyzed in 21 of 23 cases in this study; therefore, we included all cases in the analysis. In addition, we also performed subgroup analysis for the cases with tumor content ratio of 24% or more. The results were similar to those for all cases (Figure S1 and Table S1). The limitations of the current study also included the retrospective nature of our analysis and the small number of samples collected at a single center. Thus, a selection bias may have occurred. A multicenter, prospective study is needed to determine the samples that are suitable for ODxTT.

In conclusion, although there is still room for consideration regarding the specimens that are suitable for ODxTT, our results indicate that tissue samples with a tissue surface area above 1.04 mm^2^ and a tumor cell count above 375 cells might improve the analysis success rate of ODxTT. It is sometimes necessary to assess genetic alterations with a small biopsy sample, in daily practice. The criteria will help to determine which tests should be performed; ODxTT or multiple single‐gene testing.

## Disclosure

Dr Murakami reports grants from Takeda Pharmaceutical, personal fees from AstraZeneca, personal fees from Chugai Pharmaceutical, personal fees from Boehringer Ingelheim, personal fees from Taiho Pharmaceutical, personal fees from Ono Pharmaceutical. Dr Kato reports grants and personal fees from MSD, grants and personal fees from Novartis, grants and personal fees from Ono, grants and personal fees from Pfizer, grants and personal fees from Taiho, personal fees from Daiichi‐Sankyo, personal fees from F. Hoffmann‐La Roche, personal fees from Nippon Kayaku, personal fees from Nitto Denko, personal fees from Shionogi, personal fees from Sumitomo Dainippon, personal fees from Takeda, grants from Astellas, grants from Kyorin, grants from Kyowa‐Kirin, grants from Regeneron. Dr Saito reports grants from Chugai Pharmaceutical, grants from AstraZeneca, personal fees from Ono Pharmaceutical, personal fees from Nippon Boehringer Ingelheim, grants from MSD, personal fees from Novartis Pharma. The other authors report no conflicts of interest.

## Supporting information


**Table S1** Multivariate logistic regression analysis of the factors that affected the results (results of analysis focusing on cases with tumor content ratio with of 24% or more)

**Figure S1** Receiver operating characteristic curves of pathological factors (results of analysis focusing on cases with tumor content ratio with of 24% or more)
