# Supplementary material for: Tissue surface area and tumor cell count affect the success rate of the Oncomine Dx Target Test in the analysis of biopsy tissue samples
Source: Thorac Cancer. 2020 Nov 13;12(2):194–200. doi: 10.1111/1759-7714.13743 (PMC7812068; doi:10.1111/1759-7714.13743)
Supplement: Supplementary file 1 — Table S1 Multivariate logistic regression analysis of the factors that affected the results (results of analysis focusing on cases with tumor content ratio with of 24% or more) [file TCA-12-194-s001.docx]

**Supplemental table 1.** Multivariate logistic regression analysis of the factors that affected the results (Results of analysis focusing on cases with tumor content ratio with of 24% or more)

|  | Results of ODxTT (0 = success/1 = failure) | | |
| --- | --- | --- | --- |
|  | OR | 95% CI | *P* value |
| Tissue surface area (≦1.04 mm^2^ / >1.04 mm^2^) | 0.11 | 0.03-0.47 | 0.003 |
| Tumor cell count (≦375 cells / >375 cells) | 0.14 | 0.04-0.58 | 0.006 |
| Tumor cell rate (≦40% / >40%) | 1.15 | 0.28-4.72 | 0.843 |

ODxTT, Oncomine Dx Target Test; OR, odds ratio; CI, confidence interval
