# Supplementary material for: Tissue surface area and tumor cell count affect the success rate of the Oncomine Dx Target Test in the analysis of biopsy tissue samples
Source: Thorac Cancer. 2020 Nov 13;12(2):194–200. doi: 10.1111/1759-7714.13743 (PMC7812068; doi:10.1111/1759-7714.13743)

Supplemental figure 1. Receiver operating characteristic curves of pathological factors. (Results of analysis focusing on cases with tumor content ratio with of 24% or more)

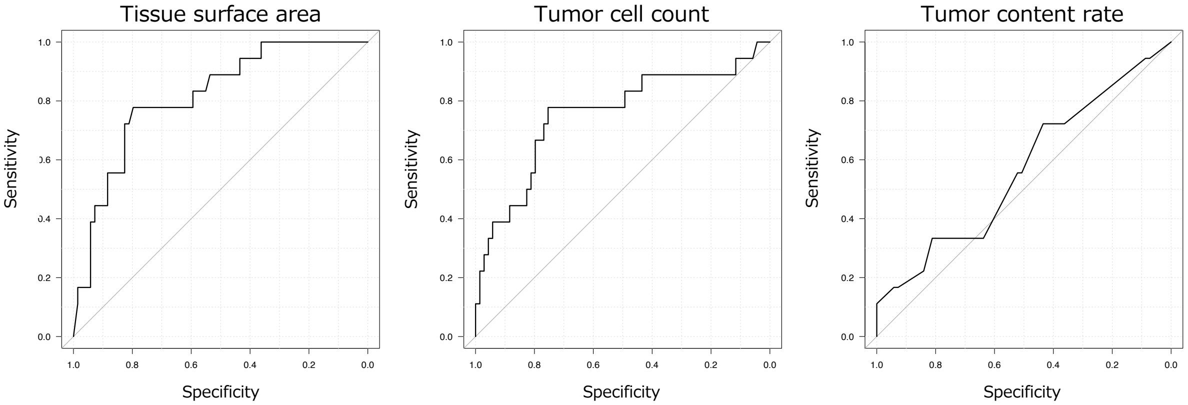

Supplement: Supplementary file 2 — Figure S1 Receiver operating characteristic curves of pathological factors (results of analysis focusing on cases with tumor content ratio with of 24% or more) [file TCA-12-194-s002.pdf]
